# Insulin Resistance Indexes as Biomarkers of Lifetime Cardiovascular Risk among Adults from Peru

**DOI:** 10.1155/2021/6633700

**Published:** 2021-03-25

**Authors:** Ricardo Rojas-Humpire, Mely Olarte-Durand, Sebastian Medina-Ramirez, Rosmery Gutierrez-Ajalcriña, Josue F. Canaza, Salomon Huancahuire-Vega

**Affiliations:** ^1^Department of Basic Sciences, Human Medicine School, Peruvian Union University (UPeU), Lima 15, Peru; ^2^P53 Research Group, Human Medicine School, Peruvian Union University (UPeU), Lima 15, Peru; ^3^Epidemiology and Environmental Health Unit, Huaycan Hospital, Lima 3, Peru

## Abstract

**Background:**

Cardiovascular disease (CVD) is the most prevalent cause of death from disease and disability in the world. Reliable markers are needed to assess and reduce cardiovascular risk. This study aimed to determine if insulin resistance indexes, triglycerides to HDL-cholesterol ratio (TG/HDL-C), and triglyceride glucose index (TyG) are biomarkers for lifetime cardiovascular risk (CVR).

**Methods:**

This analytical cross-sectional study was performed on health personnel from Huaycan Hospital in Peru. The QRISK model was used to measure lifetime CVR. The association and diagnostic accuracy for TyG calculated as Ln (TG (mg/dL) × glucose (mg/dL)/2) and TG/HDL-C ratio were determined using Poisson regression models and ROC curves with Youden index.

**Results:**

In total, 291 adults (207 women and 84 men) were analyzed. In the adjusted Poisson models, each unit of TG/HDL-C increased 1.22-fold and 1.16-fold the probability of high lifetime CVR in men and women, respectively. However, each unit of TyG increased 1.98-fold in men and 3.25-fold in women the probability of high lifetime CVR. The optimal cutoff values of TG/HDL-C were 2.64 (AUC: 0.77), 3.90 (AUC: 0.80), and 2.64 (AUC: 0.74) for the overall population, men, and women, respectively. Likewise, the optimal cutoff values of TyG were 9.04 (AUC: 0.80), 8.95 (AUC: 0.79), and 9.04 (AUC: 0.80) for the overall population, men, and women, respectively.

**Conclusion:**

TG/HDL-C and TyG presented a significant association with lifetime CVR. However, TyG presented a stronger association than TG/HDL-C. Both TG/HDL-C and TyG are shown to be reliable markers for CVR in adults.

## 1. Introduction

Cardiovascular disease (CVD) is the leading cause of death, disability, and also a major public health problem worldwide [1]. The World Health Organization (WHO) estimates that 17.5 million people died from CVD in 2014, representing 31% of all deaths recorded in the world [2]. In Peru, the CVD profile presented by the Pan American Health Organization and the WHO (PAHO/WHO) in 2014 showed that about 16% of all premature deaths for people between 30 and 69 years of age were due to CVD [3]. Early detection and prevention of CVD would help reduce the country's high mortality rate regionally [4].

The discovery and use of cardiovascular risk (CVR) biomarkers during the last few years has led to more sensitive detection methods, bringing favorable clinical outcomes to the population [5]. So far, dozens of CVR markers have been described (e.g., interleukin 6, monocyte chemotactic protein-1, tumor necrosis factor-alpha, and apolipoprotein B-100) [5–7]. The detection of most of these biomarkers requires the development of a highly sensitive technological platform. Furthermore, these are not routinely used in point-of-care testing, especially because of the high cost for most of them.

Metabolic syndrome (MetS) is the most important risk factor for CVD worldwide [8]. MetS is a cluster of metabolic disorders characterized by visceral obesity, dyslipidemia, hyperglycemia, hypertension, and a procoagulant and inflammatory state with insulin resistance (IR) as a common outcome [9]. Certain biomarkers of IR, such as triglyceride glucose index (TyG) and triglycerides to HDL-cholesterol ratio (TG/HDL-C), have shown to be useful in identifying people at high risk of developing a cardiovascular problem at an early stage [10–12]. Elevated plasma triglycerides are risk markers for CVD, type 2 diabetes, and metabolic syndrome [13]. The TG/HDL-C can help predict patients at an increased risk of cardiometabolic disease as well as identify patients who mostly need intervention [14, 15]. Likewise, the TyG has been correlated with the assessment of the insulin resistance homeostasis model (HOMA-IR) and euglycemic hyperinsulinemic clamp [16, 17]. The detection of apparently healthy individuals with IR before the onset of CVD could have clinical relevance in the prevention of CVD [14]. Additionally, the direct measurement of these IR indexes does not require complex techniques, is inexpensive, and can be performed with point-of-care testing.

Patients with CVD usually presented with three or more classical risk factors, so quantifying these factors is essential to understanding the extent of this problem [4]. Different equations and scales make it possible to quantify CVR, their differences being mainly due to the specific parameters established for the calculation and population to apply [18]. The most common risk score used in the Latin American population is the Framingham and American College of Cardiology/American Heart Association (ACC/AHA) risk score, which estimates the risk of developing myocardial infarct, stroke, or peripheral artery disease at 10 years [19]. In Peru, the prevalence of high CVR is approximately 29%; however, young, middle-aged, and older adult proportions could change the prevalence of CVR in the risk calculators at 10 years [20]. Recently, the QRISK model was developed to estimate the lifetime CVR up to 95 years, recommended for young and middle-age adult populations [21]. Based on the relationship of IR indexes (TG/HDL-C and TyG) with cardiometabolic changes, these could be practical and reliable biomarkers for CVR in primary health care. For that reason, in the present study, we explored the association and diagnostic accuracy of TG/HDL-C and TyG to lifetime CVR in adults from a Peruvian hospital.

## 2. Methods

### 2.1. Design of the Primary Study

We conducted a cross-sectional analytical study using data from the plan for the prevention and surveillance of communicable and noncommunicable diseases at the Huaycan Hospital, Lima, Peru, in 2019. In this prevention plan, clinical evaluation, self-reported questionnaires, medical images, and laboratory tests were performed to prevent and diagnose diseases in healthcare personnel. These explained that their medical data would be used for future research, and written informed consent was obtained from all participants.

This study was approved by the Ethics Committee of the Universidad Peruana Union (2020-CEUPeU-00021) and authorized by the Huaycan Hospital (031-2020) to use medical records from health personnel.

### 2.2. Inclusion and Exclusion Criteria

We included data of healthcare personnel between 20 and 79 years old of both gender from Huaycan Hospital. Participants with a medical record of myocardial infarction, coronary artery disease, cerebrovascular disease, peripheral arterial disease, as well as those who used antihyperlipidemic agents, pregnant women, participants who did not completely fill out the form, those who did not sign informed consent, and those who did not do laboratory tests were excluded.

### 2.3. Assessment of Cardiovascular Risk

The ACC/AHA risk score was calculated to measure general CVR over 10 years [22]. This score presented the best discrimination (AUC = 0.78) of general CVR in a Latin American population [19]. The population was categorized as low, borderline, intermediate, and high risk according to the ACC/AHA 2019 guidelines on the primary care of cardiovascular diseases [23].

The QRISK model was used to measure the lifetime CVR [21] in low, borderline, and intermediate risk populations based on ACC/AHA risk score, using the QRISK^®^ online calculator (https://qrisk.org/lifetime/index.php).

### 2.4. Definition of Variables

Lifetime CVR was categorized into high (≥39%) and low (<39%) risks [24]. Diabetes was defined as, diagnosed by doctor, currently taking antidiabetic medication, fasting plasma glucose ≥126 mg/dL, or HbA1c ≥6.5%. Body mass index was categorized as underweight (<18.5 kg/m^2^), normal (18.5–24.9 kg/m^2^), overweight (25–29.9 kg/m^2^), and obese (≥30 kg/m^2^). Smoker, antihypertensive medication, and alcohol consumption were assessed based on self-reported questionnaires from the plan for the prevention and surveillance of communicable and noncommunicable diseases at the Huaycan hospital.

TG/HDL-C and TyG were selected as IR indexes because they have been shown to be accurate indicators of IR diagnosis in many populations [15, 25]. Additionally, these IR indexes present accessible alternatives to HOMA-IR or glucose clamp [26, 27]. TG/HDL-C was calculated using the following formula: fasting TG (mg/dL)/fasting HDL-cholesterol (mg/dL), and analyzed such a continuous variable. TyG was calculated using the formula: Ln (fasting TG (mg/dL) × fasting plasma glucose (mg/dL)/2), and analyzed as a continuous variable.

### 2.5. Statistical Analysis

Statistical analyses were performed with R program version 4.0.2. Numerical variables were described with mean and standard deviation. Categorical variables were described in absolute and relative frequencies. To assess the association between IR indexes (TG/HDL-C and TyG) and lifetime CVR, prevalence ratios (PR) and their respective 95% confidence intervals (95% CI) were determined using Poisson regression models with robust variance. The first model examined the bivariate association between IR indexes and lifetime CVR. The second model for TG/HDL-C and TyG were adjusted for HbA1c, LDL-C, uric acid, and alcohol consumption. Receiver operating characteristic (ROC) curve analysis was applied to estimate the accuracy of IR indexes to lifetime CVR with the calculation of the area under the curve (AUC) with 95% CI. The optimal cutoff value of each marker was estimated using the Youden index analysis. A *p* value <0.05 was considered statistically significant.

## 3. Results

### 3.1. General Characteristics of the Participants

We analyzed data from 291 participants, 84 men (28.9%) and 207 women (71.1%), with an average age of 46 ± 10 years. Most of the variables of the overall population presented in Table 1 show average values in normal ranges. Overall, 49.1% of the participants were overweight, 27.1% was obese, 92.8% consume alcohol less than 20 g/day, 13.1% had diabetes, and 4.8% was smokers (≥1 cigarette/day). On the other hand, some cardiometabolic variables, such as blood pressure, waist circumference, fasting glucose, very low-density lipoprotein cholesterol (VLDL-C), high-density lipoprotein cholesterol (HDL-C), triglycerides, total cholesterol to HDL-C ratio (TC/HDL-C), TG/HDL-C, TyG, and uric acid, presented marked differences between sex.

### 3.2. Characteristics of the Participants by Lifetime Cardiovascular Risk

The study population was categorized into high and low lifetime CVR by sex. Most of the variables showed statistically significant differences (*p* < 0.01) between high and low lifetime CVR groups (Table 2). Laboratory markers and lipid profile for the high lifetime CVR group showed abnormal values, and only uric acid was in the normal interval and without significant differences in both groups.

### 3.3. Association of Insulin Resistance Indexes and Lifetime Cardiovascular Risk

All regression models were performed with Poisson regression analysis to determine the association between IR indexes and lifetime CVR (Table 3). In the nonadjusted models, each unit of TG/HDL-C increased the probability of high lifetime CVR at 26% (PR = 1.26, 95% CI: 1.17–1.40, *p* < 0.01) in overall population, 23% (PR = 1.23, 95% CI: 1.08–1.40, *p* < 0.01) in men, and 26% (PR = 1.26, 95% CI: 1.10–1.44, *p* < 0.01) in women. Similarly, each unit of TyG increased 3-fold (PR = 3.00, 95% CI: 2.16–4.16, *p* < 0.01) the probability of high lifetime CVR in overall population, 1.99-fold (PR = 1.99, 95% CI: 1.28–3.08, *p* < 0.01) in men, and 4.08-fold (PR = 4.08, 95% CI: 2.35–7.10, *p* < 0.01) in women.

In the adjusted models, the association of TG/HDL-C and high lifetime CVR was stronger in men than other groups (PR = 1.22, 95% CI: 1.05–1.42, *p* < 0.01). On the other hand, the association of TyG and lifetime CVR was stronger in women than other groups (PR = 3.25, 95% CI: 1.39–7.60, *p* < 0.01). TyG presented the strongest association in all models.

### 3.4. Diagnostic Accuracy of Insulin Resistance Indexes

**Figure 1:**
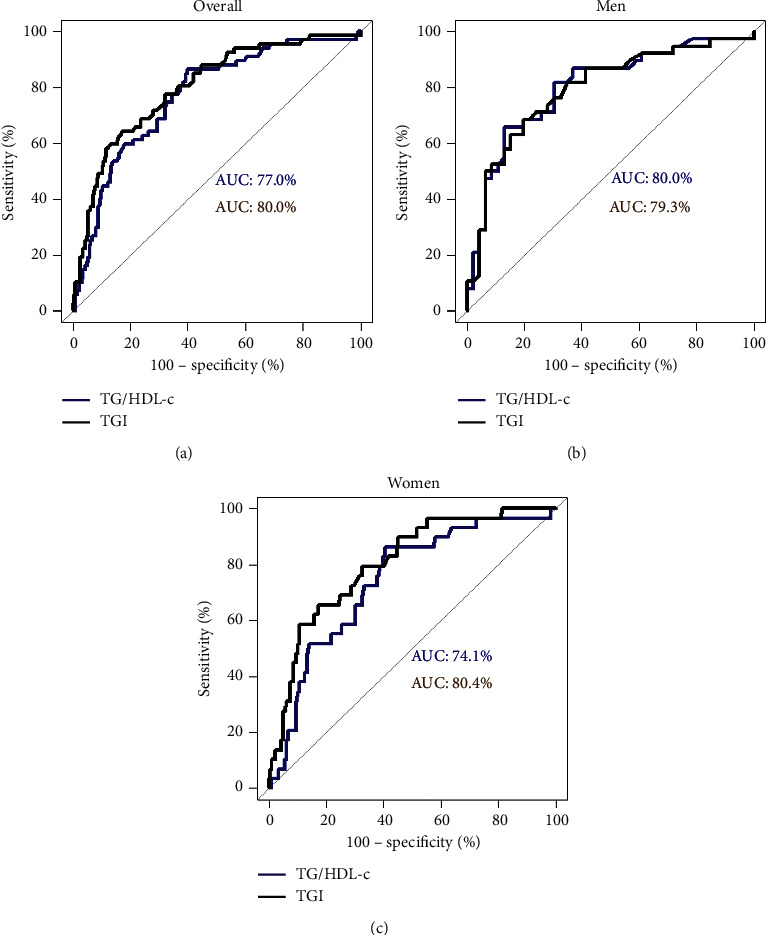
ROC curve of TG/HDL-C and TyG for predicting lifetime cardiovascular risk. (a) Overall (cutoff points by Youden index, TG/HDL-C: 2.64; TyG: 9.04). (b) Men (cutoff points by Youden index, TG/HDL-C: 3.90; TyG: 8.95). (c) Women (cutoff points by Youden index, TG/HDL-C: 2.64; TyG: 9.04).

The ROC analysis of IR indexes and lifetime CVR overall showed that the AUC of TG/HDL-C was 0.77 (sensitivity: 0.86 and specificity: 0.60) while the TyG AUC was 0.80 (sensitivity: 0.64, specificity: 0.83). TG/HDL-C in men presented an AUC of 0.80 (sensitivity: 0.66 and specificity: 0.87) while their TyG AUC was 0.79 (sensitivity: 0.69, specificity: 0.80). TG/HDL-C in women showed an AUC of 0.74 (sensitivity: 0.86 and specificity: 0.60) while their TyG AUC was 0.80 (sensitivity: 0.66, specificity: 0.82) (Figure 1). Each marker presented good diagnostic accuracy to high lifetime CVR in men, women, and overall. The optimal cutoff point from Youden index analysis of each marker to predict high lifetime CVR is presented in Table 4.

## 4. Discussion

The identification of seemingly healthy people with IR before the manifestation of CVD is an important step in the prevention of cardiovascular events. Several studies widely use TG/HDL-C and TyG indices as markers of IR [26, 28–30]. Recent studies mention that these indexes could be useful to identify people at high risk of developing some cardiovascular events early [14, 15, 31–33]. In the present study, TG/HDL-C and TyG indices presented an independent association to lifetime CVR and good diagnostic accuracy. To our knowledge, this is the first study to assess the association of IR indexes to lifetime CVR in a Peruvian population.

Several studies showed that TG/HDL-C is a risk factor for global cardiovascular mortality [25], calcification of arteries [34], and coronary artery disease [35] in adult and young population [11, 36]. For example, a study in adults from the U.S. found that high TG/HDL-C (>2.5 in women or >3.5 in men) increased by 1.5-fold the incidence of type 2 diabetes and CVD [14]. Another study showed that high TG/HDL-C increased by 2-fold the incidence of events and mortality due to CVD in nondiabetic dialysis patient from Taiwan [37], and in obese type 2 diabetes patients, TG/HDL-C ≥1.9 presented a 1.6-fold increase in the risk of CVD compared with normal weight patients [36]. In the present study, the TG/HDL-C ratio increased 1.22-fold and 1.26-fold the high lifetime CVR in men and women, respectively, according to the adjusted model. The relationship could be explained through increased IR when TG/HDL-C is high [25].

TyG is a surrogate marker of IR that in different studies was proposed as a predictor of CVD [38]. Likewise, a study in patients from Taiwan showed that high levels of TyG increased by 1.5-fold the risk of CVD [7]. Another study from Spain found that high levels of TyG increased by 2-fold the incidence of CVD in adults, and the ROC models integrating TyG were better than the Framingham model [15]. In the same way, a study in children and adolescents with normal weight from Mexico showed that the highest quintile of TyG was associated with hypertriglyceridemia, hyperglycemia, and low HDL-C [39]. We found that TyG significantly increases by 1.98-fold and 3.25-fold the high lifetime CVR in men and women, respectively. Additionally, the cutoff values of TG/HDL-C and TyG for lifetime CVR in our study were similar to other studies [32, 40], but cutoff values for IR slightly differ of the CVR [17, 41]. This could be because IR is the beginning of metabolic changes that predispose many cardiometabolic diseases [9].

In this study, both TG/HDL-C and TyG presented a good AUC for lifetime CVR. Likewise, studies in Asian population showed that the cutoff value of TyG was 8-9 with an AUC of 0.6–0.8 for predicting cardiovascular events [15, 38, 42]. On the other hand, a study in a Japanese population determined that the optimal cutoff values for TG/HDL-C were 2.9 for men and 2.3 for women with an AUC of 0.8 for predicting cardiometabolic risk [43]. In the same way, a population from China reported TG/HDL-C cutoff values of 3.22 for men and 2.3 for women (converted to mg/dL from mmol/L) for predicting elevated CVR factors [44]. Another study in Argentinian population showed that AUC of TG/HDL-C was 0.85 with cutoff values of 3.1 for men and 2.2 for women, while AUC of TyG was 0.88 with cutoff values of 8.8 for men and 8.7 for women, both for IR predicting [45]. However, a study in diabetic and nondiabetic populations from Mexico showed that cutoff of TyG was 4.68 for IR with AUC of 0.86; this high cutoff value can be explained by the prevalence of diabetes and prediabetes in the study [16]. It is important to determine the optimal cutoff values for TyG and TG/HDL-C for predicting cardiovascular events in a Latin American population.

IR is a metabolic state that leads to many cardiometabolic diseases [9]. This is caused through defects in the metabolic pathway of insulin receptors, mammalian target of rapamycin (mTOR), and ribosomal protein S6 kinase beta-1 (S6K1) with an increase of proinflammatory proteins, endothelial dysfunction, the renin-angiotensin system, and dyslipidemia [44]. Additionally, this metabolic state is related to cultural lifestyle factors such as nutrition and physical activity [46]. In our study, we found adults with normal traditional metabolic markers (Table 1) but with high lifetime CVR; this fact highlights the necessity of new markers in medical practice and primary prevention in cardiovascular health.

The prediction of lifetime CVR in developing countries such as Peru is very important given all the consequences of CVD in the population and the healthcare system. TG/HDL-C and TyG are inexpensive and accessible markers. In this way, they could aid in the assessment of CVR and establish therapeutic goals in primary prevention care.

This study presents some limitations, notably that it was carried out in health personnel from a hospital from Peru with high prevalence of women due to predominance of women in some health professions such as nurses, midwives, and healthcare technicians in Peru, and for that reason, the study cannot be generalized for the general population. There are no cardiovascular risk scores developed specifically for the Latin American population, but the AHA/ACC risk score is the most accurate for this population. It was not possible to assess causality of variables due to the nature of the cross-sectional study design used. The findings are also limited by the absence of assessment for hypothyroidism and renal and liver diseases. However, no thyroid, renal, or liver pathologies were registered in the participants' medical records. In this study, we used CVR calculators (AHA/ACC risk score and QRISK model) that integrate many classical CVR factors adjusted in predictive models. TG/HDL-C and TyG were adjusted by potential confounders not included in CVR calculators used; however, future studies must consider these limitations and include more lifestyle factors.

## 5. Conclusions

In conclusion, the TG/HDL-C and TyG showed a significant independent association to lifetime CVR after adjusted potential confounders in adults from Peru. The association of TyG was stronger than TG/HDL-C in this study. Likewise, diagnostic accuracy of both markers was good in men, women, and overall population. These indexes are practical and reliable markers for CVR and useful tools in primary prevention programs for cardiovascular health.

## Figures and Tables

**Table 1:** General characteristics of participants by sex.

Variables	Overall (*n* = 291)	Men (*n* = 84)	Women (*n* = 207)	*p* value
Age (years)	46 ± 10	47 ± 10	45 ± 10	0.129
Weight (kg)	67.8 ± 13.0	75.4 ± 13.9	64.8 ± 11.3	<0.001
SBP (mmHg)	108 ± 13	114 ± 13	106 ± 12	<0.001
DBP (mmHg)	69 ± 10	73 ± 12	67 ± 10	<0.001
WC (cm)	91.8 ± 10.7	96.0 ± 12.3	90.1 ± 9.6	<0.001
Glucose (mg/dL)	95.9 ± 32.8	100.8 ± 33.8	93.9 ± 32.3	0.001
HbA1c (%)	6.02 ± 1.03	6.10 ± 1.26	5.99 ± 0.92	0.890
Cholesterol (mg/dL)	192.9 ± 36.7	196.8 ± 37.9	191.3 ± 36.2	0.170
LDL-C (mg/dL)	114.6 ± 30.2	118.9 ± 31.4	112.8 ± 29.6	0.061
VLDL-C (mg/dL)	28.7 ± 12.7	31.4 ± 14.1	27.6 ± 12.0	0.035
HDL-C (mg/dL)	49.9 ± 10.3	47.18 ± 8.38	51.06 ± 10.82	0.003
Triglycerides (mg/dL)	151.1 ± 77.5	170.8 ± 92.2	143.1 ± 69.3	0.030
Chol/HDL-C	3.99 ± 1.03	4.24 ± 0.88	3.89 ± 1.08	<0.001
TG/HDL-C	3.20 ± 1.91	3.70 ± 2.06	3.00 ± 1.81	0.003
TyG	8.74 ± 0.58	8.89 ± 0.64	8.68 ± 0.54	0.004
Uric acid (mg/dL)	3.8 ± 1.0	4.7 ± 0.9	3.5 ± 0.9	<0.001
Smoker^a^				
≥1 cigarette/day	14 (4.8)	9 (4.3)	5 (6.0)	0.782
<1 cigarette/day	277 (95.2)	198 (95.7)	79 (94.0)	
Diabetes^a^				
Yes	38 (13.1)	28 (13.5)	10 (11.9)	0.857
No	253 (86.9)	179 (86.5)	74 (88.1)	
BMI^a^				
Normal	69 (23.7)	52 (25.1)	17 (20.2)	0.573
Overweight	143 (49.1)	98 (47.3)	45 (53.6)	
Obesity	79 (27.1)	57 (27.5)	22 (26.2)	
Alcohol consumption^a^				
<20 g/day	270 (92.8)	194 (93.7)	76 (90.5)	0.472
≥20 g/day	21 (7.2)	13 (6.3)	8 (9.5)	

Data expressed as mean ± standard deviation or number (%). SBP, systolic blood pressure; DBP, diastolic blood pressure; WC, waist circumference; HbA1c, glycated hemoglobin; LDL-C, low-density lipoproteins cholesterol; VLDL-C, very low-density lipoproteins cholesterol; HDL-C, high-density lipoproteins cholesterol; Chol/HDL-C, total cholesterol to HDL-cholesterol ratio; TG/HDL-C, triglycerides to HDL-cholesterol ratio; TyG, triglyceride glucose index; BMI, body mass index. ^a^Prevalence.

**Table 2:** Cardiometabolic variables of participants by lifetime cardiovascular risk and sex.

Variables	Men		*p* value	Women		*p* value
	High^a^ (*n* = 38)	Low^a^ (*n* = 46)		High^a^ (*n* = 29)	Low^a^ (*n* = 178)	
Age (years)	47 ± 9	48 ± 11	0.668	52 ± 8	44 ± 10	0.001
Weight (kg)	82.8 ± 15.1	69.2 ± 9.1	<0.001	72.7 ± 10.8	63.5 ± 10.8	<0.001
SBP (mmHg)	118 ± 11	111 ± 13	0.010	114 ± 13	104 ± 12	<0.001
DBP (mmHg)	77 ± 12	71 ± 11	0.014	71 ± 9	66 ± 9	0.007
BMI (kg/m^2^)	30.5 ± 4.7	26.4 ± 2.5	<0.001	31.2 ± 4.6	27.4 ± 4.0	<0.001
WC (cm)	100.4 ± 15.5	92.3 ± 7.0	0.002	96.7 ± 9.2	89.0 ± 9.2	<0.001
Glucose (mg/dL)	111.8 ± 47.3	91.7 ± 8.8	0.006	120.4 ± 75.8	89.6 ± 13.1	<0.001
HbA1c (%)	6.47 ± 1.78	5.78 ± 0.29	0.012	6.86 ± 1.56	5.85 ± 0.67	<0.001
Cholesterol (mg/dL)	213.0 ± 35.3	183.5 ± 34.8	<0.001	221.6 ± 39.9	186.4 ± 33.1	<0.001
LDL-C (mg/dL)	133.3 ± 28.1	107.0 ± 29.1	<0.001	138.2 ± 40.4	108.6 ± 25.3	<0.001
VLDL-C (mg/dL)	36.3 ± 12.6	27.4 ± 14.2	0.003	35.0 ± 11.0	26.4 ± 11.7	<0.001
HDL-C (mg/dL)	44.9 ± 8.4	49.1 ± 7.9	0.021	48.3 ± 10.2	51.5 ± 10.9	0.143
Triglycerides (mg/dL)	211.9 ± 99.0	136.8 ± 70.9	<0.001	188.1 ± 67.5	135.8 ± 67.0	<0.001
Chol/HDL-C	4.84 ± 0.86	3.75 ± 0.53	<0.001	4.73 ± 1.12	3.75 ± 1.01	<0.001
TG/HDL-C	4.79 ± 2.23	2.81 ± 1.38	<0.001	4.13 ± 1.80	2.82 ± 1.75	<0.001
TyG	9.20 ± 0.69	8.64 ± 0.46	<0.001	9.17 ± 0.50	8.60 ± 0.50	<0.001
Uric acid (mg/dL)	4.8 ± 0.9	4.6 ± 0.8	0.284	3.7 ± 0.8	3.4 ± 0.9	0.114

Data expressed as mean ± standard deviation. SBP, systolic blood pressure; DBP, diastolic blood pressure; BMI, body mass index; WC, waist circumference; HbA1c, glycated hemoglobin; LDL-C, low-density lipoproteins; VLDL-C, very low-density lipoproteins cholesterol; HDL-C, high-density lipoproteins cholesterol; Chol/HDL-C, total cholesterol to HDL-cholesterol ratio; TG/HDL-C, triglycerides to HDL-cholesterol ratio; TyG, triglyceride glucose index. ^a^Lifetime cardiovascular risk.

**Table 3:** Prevalence ratio and 95% CIs for lifetime cardiovascular risk according to levels of insulin resistance indexes.

Markers	Overall	Men	Women
	PR (95% CI)	PR (95% CI)	PR (95% CI)
TG/HDL-C			
Model 1^a^	1.26 (1.17–1.40) ^*∗∗*^	1.23 (1.08–1.40) ^*∗∗*^	1.26 (1.10–1.44) ^*∗∗*^
Model 2^b^	1.15 (1.04–1.28) ^*∗∗*^	1.22 (1.05–1.42) ^*∗∗*^	1.16 (1.04–1.36) ^*∗*^
TyG			
Model 1^a^	3.00 (2.16–4.16) ^*∗∗*^	1.99 (1.28–3.08) ^*∗∗*^	4.08 (2.35–7.10) ^*∗∗*^
Model 2^b^	2.47 (1.44–4.23) ^*∗∗*^	1.98 (1.01–3.87) ^*∗*^	3.25 (1.39–7.60) ^*∗∗*^

PR, prevalence ratio; 95% CI, 95% confidence interval; TG/HDL-C, triglycerides to HDL-cholesterol ratio; TyG, triglyceride glucose index. ^a^Nonadjusted and ^b^adjusted for HbA1c, LDL-C, uric acid, and alcohol consumption;  ^*∗*^*p* < 0.05;  ^*∗∗*^*p* < 0.01.

**Table 4:** Diagnostic accuracy of TG/HDL-C and TyG in predicting lifetime cardiovascular risk and their optimal cutoff values.

Predictors	Overall	Men	Women
	Value (CI 95%)	Value (CI 95%)	Value (CI 95%)
TG/HDL-C			
Optimal cutoff^a^	2.64	3.90	2.64
AUC	0.77 (0.71–0.83)	0.80 (0.70–0.90)	0.74 (0.65–0.83)
Sensitivity	0.86 (0.76–0.94)	0.66 (0.49–0.80)	0.86 (0.68–0.96)
Specificity	0.60 (0.54–0.67)	0.87 (0.74–0.95)	0.60 (0.52–0.67)
PPV	0.39 (0.33–0.60)	0.81 (0.64–0.90)	0.26 (0.20–0.58)
NPV	0.94 (0.88–0.95)	0.75 (0.60–0.90)	0.96 (0.90–0.97)

TyG			
Optimal cutoff^a^	9.04	8.95	9.04
AUC	0.80 (0.74–0.86)	0.79 (0.69–0.89)	0.80 (0.72–0.88)
Sensitivity	0.64 (0.51–0.76)	0.68 (0.51–0.82)	0.66 (0.46–0.82)
Specificity	0.83 (0.77–0.88)	0.80 (0.66–0.91)	0.82 (0.76–0.88)
PPV	0.53 (0.44–0.66)	0.74 (0.58–0.86)	0.38 (0.29–0.60)
NPV	0.88 (0.82–0.92)	0.76 (0.60–0.88)	0.94 (0.87–0.96)

AUC, area under the curve; TG/HDL-C, triglycerides to HDL-cholesterol ratio; TyG, triglyceride glucose index; PPV, positive predictive value; NPV, negative predictive value.

## Data Availability

The datasets used and analyzed for this study are available from the corresponding author upon reasonable request.
